# Investigation of the Relationship between the Mid_Thigh Adipose Tissue Distribution Measured by MRI and Serum Osteocalcin—A Sex-Based Approach

**DOI:** 10.3390/nu14010112

**Published:** 2021-12-27

**Authors:** Eva Hassler, Gunter Almer, Gernot Reishofer, Hannes Deutschmann, Wilfried Renner, Markus Herrmann, Stefan Leber, Alexander Staszewski, Felix Gunzer, Harald Mangge

**Affiliations:** 1 Division of Neuroradiology, Vascular and Interventional Radiology, Department of Radiology, Medical University Graz, 8036 Graz, Austria; eva.hassler@medunigraz.at (E.H.); hannes.deutschmann@medunigraz.at (H.D.); stefan.leber@medunigraz.at (S.L.); alexander.staszewski@stud.medunigraz.at (A.S.); felix.gunzer@medunigraz.at (F.G.); 2 Clinical Institute of Medical and Chemical Laboratory Diagnostics, Medical University Graz, 8036 Graz, Austria; gunter.almer@medunigraz.at (G.A.); wilfried.renner@medunigraz.at (W.R.); markus.herrmann@medunigraz.at (M.H.); harald.mangge@medunigraz.at (H.M.); 3 Department of Radiology, Medical University Graz, 8036 Graz, Austria; 4 Biotech Med Graz, 8010 Graz, Austria

**Keywords:** osteocalcin, intramuscular adipose tissue, subcutaneous adipose tissue, glucose metabolism, insulin

## Abstract

Osteocalcin, in its non-carboxylated form, has a positive effect on glucose metabolism. Additionally, osteocalcin levels are related to body composition, especially muscle mass. The relation to the distribution of different adipose tissue types, such as subcutaneous, intermuscular, and visceral adipose tissue, is unclear. This study aimed to investigate associations between serum osteocalcin and the distribution of subcutaneous and intermuscular adipose tissue of the mid-thigh. Furthermore, the influence of different training methods on osteocalcin levels was investigated. We performed adipose tissue quantification of subcutaneous adipose tissue (SAT) and intramuscular adipose tissue (IMAT) using MRI measurements of the mid-thigh in 128 volunteers (63 male/65 female). Laboratory analysis included blood lipid panel, serum insulin, adiponectin, and osteocalcin measurements. The main observation was a significant correlation of total serum osteocalcin (TOC) and the distribution of adipose tissue of the mid-thigh (SAT/(SAT + IMAT)) (cc = −0.29/*p*-value = 0.002), as well as the cross-sectional muscle area (MA), increasing with the weekly resistance training duration in males. Additionally, TOC (*p*-value = 0.01) and MA (*p*-value = 0.03) were negatively related to serum insulin. The significant relationship between TOC and SAT/(SAT + IMAT) is a new finding and confirms the negative influence of IMAT on glucose metabolism in a sex-specific approach. We could substantiate this by the negative relation of TOC with serum insulin.

## 1. Introduction

Metabolic syndrome, which represents a complex picture of heterogeneous pathophysiologic backgrounds, is on a worldwide rise. It is associated with an increased risk of cardiovascular diseases, such as stroke and heart attack, and metabolic conditions, such as type 2 diabetes [1,2]. Studies have shown that metabolism, especially glucose metabolism, can be influenced by bone tissue’s structural and endocrine properties. Bone tissue is not only an important component of the musculoskeletal system with supporting and protective functions but has also received attention as an important endocrine organ [3,4,5]. 

Both fibroblast growth factor (FGF) and uncarboxylated osteocalcin (ucOC) are produced in bone tissue, and osteocalcin, in particular, seems to have an important influence on glucose metabolism [4,6,7]. The effect of ucOC on insulin secretion is mediated predominantly by glucagon-like peptide-1 (GLP-1), released from intestinal endocrine cells [8]. Apart from this, osteocalcin levels influence male fertility and brain development [7,9]. This supports the thesis that osteocalcin is a marker for bone remodeling and seems to play an important hormonal role [10]. The known antidiabetic effects of osteocalcin are based on different mechanisms—firstly, via its direct action on the beta cells of the pancreatic islets by the uncarboxylated form, and secondly, by indirectly promoting the production of adiponectin in adipocytes [11].

**Figure 1:**
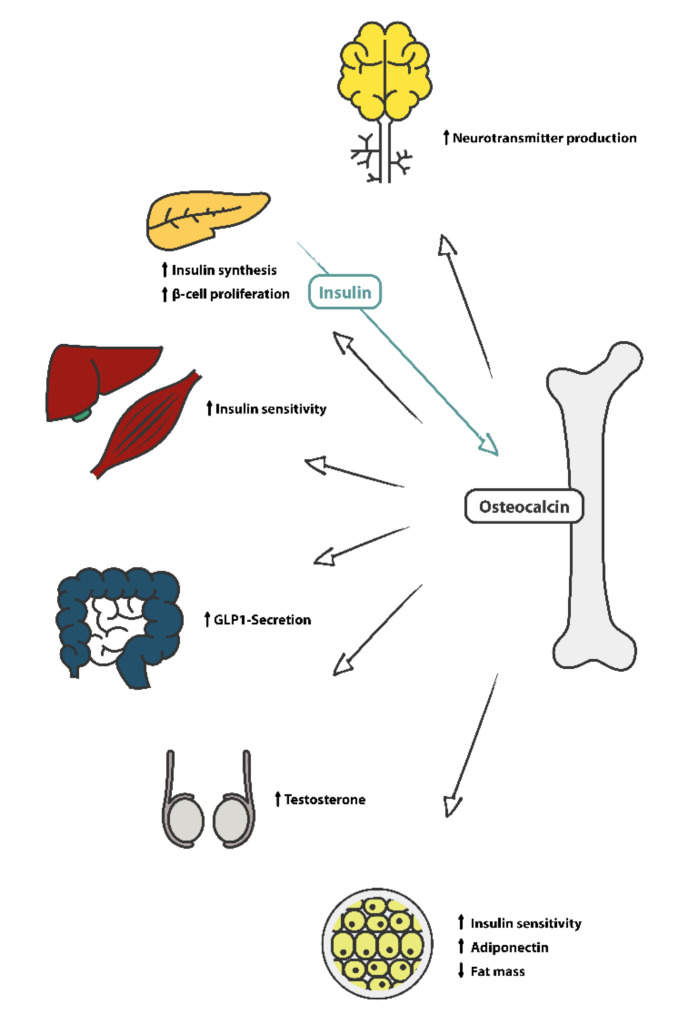
Overview of the different known effects of serum osteocalcin.

Further, it was found that osteocalcin causes an increased breakdown of body fat deposits [12]. Animal studies revealed the mechanism of action for osteocalcin in the amelioration of insulin resistance by decreasing inflammation and improving insulin signaling in white adipose tissue over the expression of Slc2a4/GLUT4 [13]. A pictorial overview of the known effects of serum osteocalcin is shown in Figure 1. Some studies have shown a relationship between body composition and serum osteocalcin levels, particularly a positive association with muscle mass. Additionally, osteocalcin apparently may counteract age-related muscle loss [7,14].

An important sign of age- and inactivity-related muscle loss is the increasing infiltration of muscle tissue by adipose tissue in the whole body. This infiltrative adipose tissue in the muscles, usually referred to as intramuscular adipose tissue (IMAT), is defined as the adipose tissue between the muscle fibers within the fascia. In recent years, a strong association between IMAT and cardiovascular risk factors has emerged. Especially, its influence on glucose has been highlighted in several studies [15,16,17]. Furthermore, not only the total amount of IMAT but also its local distribution seems to play an important role. IMAT in the thigh muscles was more strongly associated with insulin resistance and cardiovascular risk factors than IMAT in the muscle tissue of the calf. IMAT may also cause reduced blood flow and reduced insulin diffusion capacity in the muscle tissue [18]. However, the exact mechanisms of how IMAT relates to insulin resistance remain unclear; it is assumed that an increased local free fatty acid (FAA) concentration might play a role [19]. A previous study of our group showed a significant positive correlation of serum adiponectin with the adipose tissue distribution of the mid-thigh represented through the ratio between subcutaneous adipose tissue (SAT) and IMAT in both sex groups [20]. Additionally, it is known that serum adiponectin has a beneficial effect on glucose metabolism [21].

Magnetic resonance imaging (MRI) allows for the noninvasive investigation of body composition, quantifies different adipose tissue compartments, and is considered the gold standard. Additionally, MRI enables quantification of adipose tissue infiltration in the skeletal muscle and is the only method to assess intramuscular adipose tissue distribution without exposure to ionizing radiation [15,22,23].

Previous studies have already shown that exercise, especially strength training, is an important preventive measure to reduce age-related changes in muscle tissue as well as the loss of muscle mass (MM) and the resulting negative effects on muscle-cell and glucose metabolism [24,25]. In addition, a recent meta-analysis also indicated an effect of exercise interventions on total osteocalcin levels, uncarboxylated osteocalcin, and serum adiponectin levels. 

Since osteocalcin affects glucose metabolism, and intermuscular adipose tissue of the mid-thigh appears to be related to glucose metabolism, our study aimed to investigate whether the distribution of adipose tissue of the mid-thigh (SAT/(SAT + IMAT)) is related to total serum osteocalcin levels (TOC).

Furthermore, we paid attention to possible differences between male and female groups. In addition, in terms of preventing cardiovascular disease and age-related muscle loss, we wanted to elucidate whether and which form of physical activity could affect osteocalcin levels and influence the MA and the ratio SAT/(SAT + IMAT) of the thigh in a positive way.

## 2. Materials and Methods

### 2.1. Subjects

The study was approved by the local ethics committee (EK-Nr. 29-585 ex 16/17). Written informed consent was obtained from all participants before enrolment. We took blood samples and used data from a part of the cohort as recruited from our MRI-fatty-tissue-measurement study [20]. In this cross-sectional study, we included 128 subjects (63 males, 65 females, age: 44.10 ± 11.10 (mean ± SD years)). Prior to the study, volunteers with known lipid disorders; metabolic disorders, such as diabetes, muscle diseases, neurological disorders, or chronic diseases; as well as a regular intake of cholesterol-reducing medication or hormones, were excluded. Due to MRI examination, all patients with metallic implants and/or claustrophobia could not be included in the study. We obtained anthropometric measurements in every volunteer, including height, waist, and hip circumferences, using an inelastic measuring tape. All volunteers were weighted using the same personal scale to avoid bias. The parameters waist/hip ratio and waist/height ratio were calculated using MS Office Excel (Office 365, Microsoft Cooperation, One Microsoft Way, Redmond, WA, USA).

A questionnaire on pre-existing conditions and lifestyle was answered by all enrolled study participants. They indicated the frequency, duration, and nature of their sporting activities. The weekly training frequency and duration for both resistance and endurance training were collected.

### 2.2. Magnetic Resonance Imaging (MRI)

The Dixon technique is a fast and frequently used method for examining adipose tissue. A water-only (W) and fat-only signal (F) is generated due to the different magnetic properties of water and fat protons. This is also known as chemical shift imaging, which is based on a difference in the precession frequencies of the protons in the magnetic field. This allows separating fatty tissue from water-containing tissue (e.g., muscle tissue) [26].

**Figure 2:**
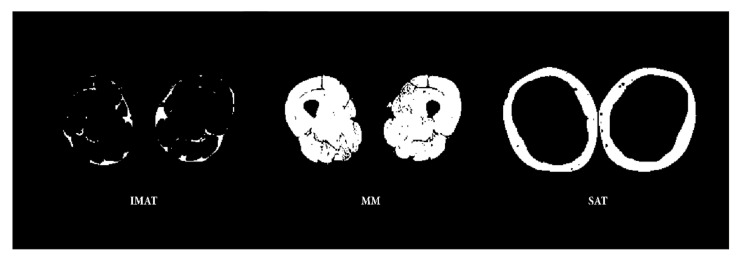
The segmentation results for IMAT, cross-sectional MA, and SAT of the mid-thigh.

To ensure comparability of the region for every subject measured with MRI, a marker was positioned at the front of the thigh. We used a standardized method developed for ultrasound measurements of the subcutaneous adipose tissue to set the marker position [27]. Afterward, we performed MRI examination of the thigh in a supine position using a 3 Tesla magnetic resonance scanner (Siemens MAGNETOM^®^ Prisma fit, Siemens Healthineers, Erlangen, Germany). The MRI protocol consists of a 2- Point DIXON Sequence (TR 4.66 ms, TE1 1.24 ms, TE2 2.47 ms, Flip angle 9°, base resolution 288, ISO Voxel Size: 1.4 × 1.4 × 1.4 mm, PAT 4), as shown in Figure 2.

### 2.3. Image Processing

MRI data were analyzed using FIJI, an open-source image processing package based on ImageJ [28]. The slice with the marker position was used for the manual segmentation of SAT and IMAT. The cross-sectional water-containing muscle area (MA) was defined. A binary mask for IMAT and SAT was generated and applied on a Dixon fat image. The amount of IMAT and SAT was measured in cm^2^. The mask for the MA was applied on Dixon fat images. We calculated the total MA and the percentage of IMAT between the MA. Additionally, we calculated the percentage of SAT in the total adipose thigh tissue as follows: SAT/(SAT + IMAT). Segmentation results for IMAT and muscle cross-sectional area (MA) and SAT are indicated in Figure 2.

### 2.4. Laboratory Analysis

Venous blood samples were collected from every subject prior to MRI measurements. The blood samples were collected after an overnight fasting period of 12 h hours. The serum lipid analyses contained total blood cholesterol (mg/dL), HDL (mg/dL), LDL (mg/dL), and serum triglycerides(mg/dL). The total cholesterol/HDL quotient was calculated using MS Office Excel 2016^®^ (Microsoft Cooperation, Microsoft Cooperation, One Microsoft Way, Redmond, WA, USA). Serum osteocalcin (mg/dL) and insulin levels (µlU/mL), and serum adiponectin levels (µg/mL), were analyzed using Human ELISA Kits^®^ (BioVendor, Brno, Czech Republic) according to the manufacturer instructions. Standards, controls (high and low), and samples were measured in duplicates. The standard curve was calculated using the four-parameter algorithm. All blood samples were analyzed in the same laboratory with a standardized protocol using frozen serum (−80 °C)**.**

### 2.5. Statistics

Statistics were performed using RStudio—R version 4.0.2 (Integrated Development for R. RStudio, PBC, Boston, MA, USA). Sex differences between the male and female groups were evaluated via Welch’s *t*-test, a modified Student’s *t*-test.

Statistical comparison of parametric continuous subject characteristics was accomplished by the 2-sided Student’s *t*-test with Pearson’s linear correlation coefficients separated in sex groups. Distributions were tested for normality using the Shapiro–Wilk test. A *p*-value < 0.05 was considered statistically significant.

## 3. Results

### 3.1. Serum and Anthropometric Parameters

In total, 128 healthy subjects (63 male, 65 female, age: females (46.12 ± 10.35 (mean ± SD))/males (43.01 ± 11.59 (mean ± SD) years) were included in the study. Table 1 shows the anthropometric measurements, height, weight, waist, and hip circumferences; the muscle areas and areas of adipose tissue compartments; and the ratios of the fat compartments; and laboratory parameters (total serum OC, lipid profile and adipokines) of the entire study population. The parameters were indicated for both sex groups.

Serum osteocalcin levels were significantly higher in the male group (8.88 ± 3.22 mg/dL (mean ± SD)) than in females (6.98 ± 3.07 mg/dL (mean ± SD)/*p*-value = 0.002). Additionally, the muscle cross-sectional area showed significantly higher values in the male group (336.18 ± 54.15 cm^2^ (mean ± SD)) than in the female group (230.67 ± 29.13 (mean ± SD)/*p*-value < 0.001). The percentage of SAT of the whole AT of the thigh shows no statistically significant difference between men (0.87 ± 0.05 (mean ± SD)) and women (0.88 ± 0.05 (mean ± SD)/*p*-value = 0.05) (Table 1).

### 3.2. Associations of Serum Osteocalcin Are Male-Related

Table 2 shows the correlation between serum osteocalcin levels and the mid-thigh adipose tissue segmentation results, including adipokines in the entire study population after segmentation and the sex-separated evaluation.

The main observation in our study was a significant positive correlation of total serum OC level with the cross-sectional MA of the thigh in the male group (cc = 0.24/*p* = 0.003) but not in the female group (cc = −0.09/*p* = *0*.49). A relation of serum OC with the distribution of adipose tissue of the mid-thigh (SAT/(SAT + IMAT)) was also only significant in the male group (cc = −0.27/*p*-value = 0.03). The MA/IMAT ratio did not show a significant relationship with TOC. Osteocalcin levels were related to the person’s age in the male study population (cc = 0.31/*p*-value = 0.02), but not in females (cc = −0.18/*p*-value = 0.15).

Another important result was the significant negative relation of serum insulin levels with serum ostelcalcin (cc = −0.32/*p*-value = 0.01) and cross-sectional MA (cc = −0.28/*p*-value = 0.03) in the male group, but not in females.

Blood lipid parameters and serum adiponectin level did not relate significantly to MA and serum osteocalcin in both sex groups.

Serum osteocalcin levels showed significantly higher levels in the male group than in females in our study population.

**Figure 3:**
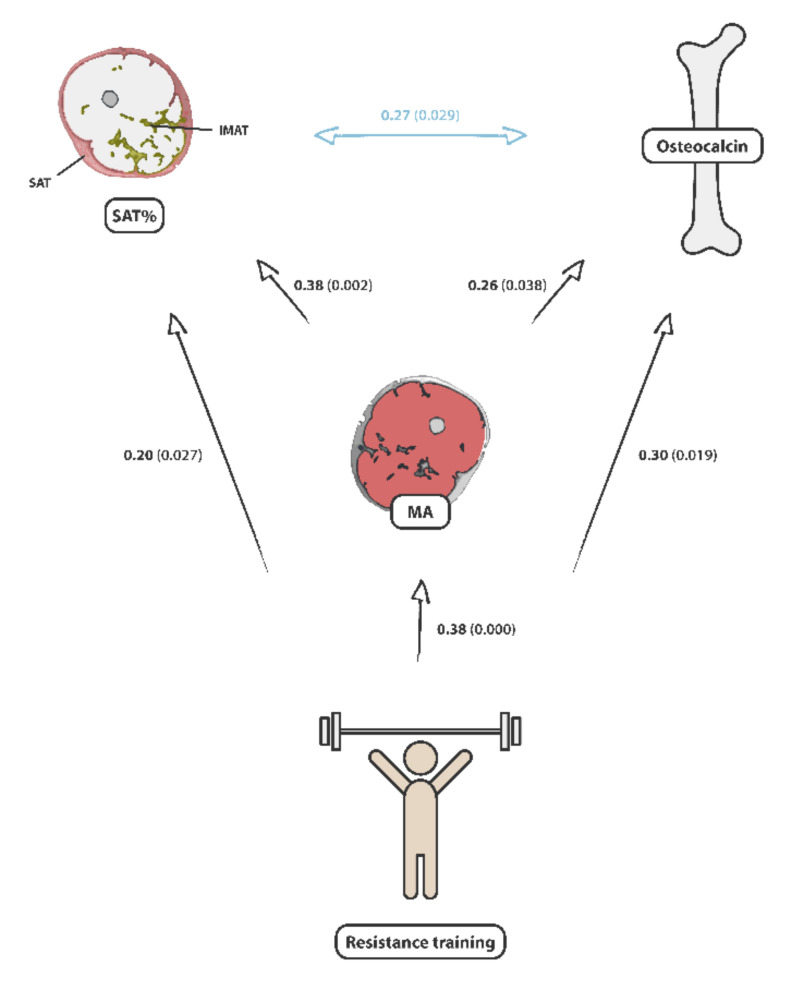
Overview of the interrelationships between SAT/(SAT + IMAT), cross-sectional muscle area, serum osteocalcin, and resistance training. The strength of the relationships is given by Pearson’s correlation coefficients and the corresponding *p*-values (cc (*p*-value)).

We found a highly significant relationship of the weekly duration of strength training in males between TOC (cc = 0.30/*p*-value = 0.019) and MA (cc = 0.38/*p*-value < 0.01) but not in females in a sex-stratified analysis. The weekly strength training frequency was also related to osteocalcin level in the male population (cc = 0.26/*p*-value = 0.014) but not in females (S.1). Interrelationships between muscle area, osteocalcin, adipose tissue, and resistance training are shown in Figure 3.

**Figure 4:**
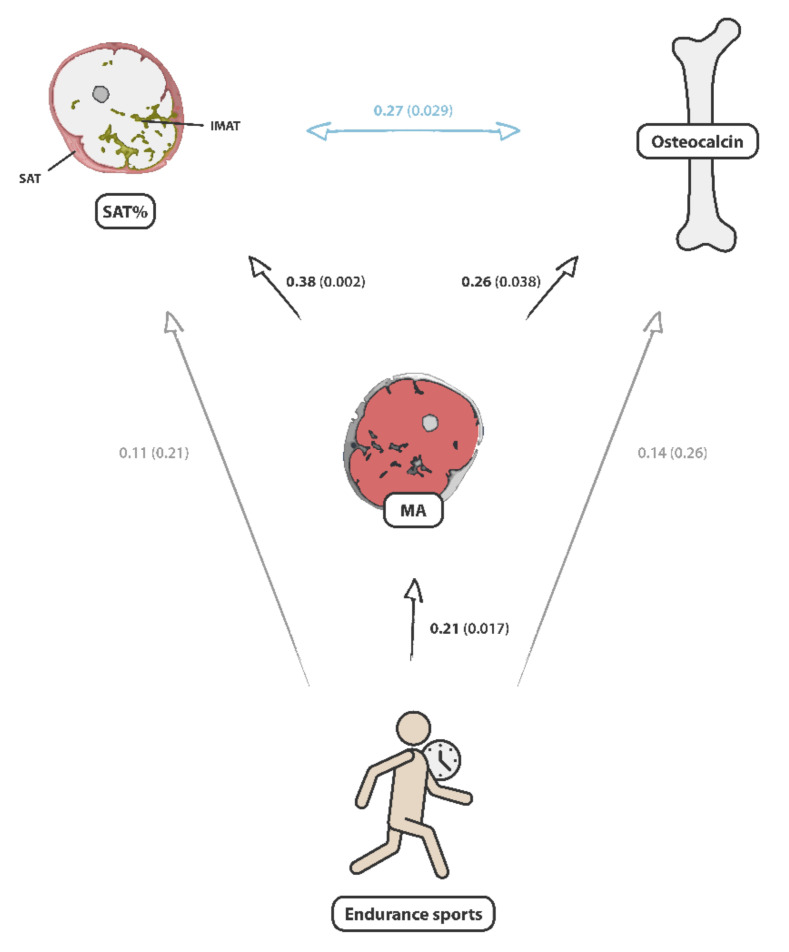
Overview of the interrelationships between SAT/(SAT + IMAT), cross-sectional muscle area, serum osteocalcin, and endurance training. The strength of relationships is given by the Pearson’s correlation coefficients and the corresponding *p*-values (cc (*p*-value)).

No correlation was found between TOC and the weekly duration of endurance training in both sex groups, but a correlation between the weekly duration of endurance sports and MA in the male group (cc = 0.21/*p* = *0*.017). Interrelations between endurance sports, muscle area, osteocalcin, and adipose tissue distribution in the mid-thigh are shown in Figure 4.

## 4. Discussion

In our study, we found a highly significant correlation of serum TOC with the cross-sectional muscle area of the mid-thigh in the male population and, further, a significant correlation of the adipose tissue distribution of the mid-thigh with TOC in healthy males but not in female adults. A relation of muscle mass with TOC had been described in former studies, but a connection with the distribution of thigh adipose tissue has not been investigated so far.

The connection of the ratio SAT/(SAT + IMAT), describing the adipose tissue distribution in the mid-thigh with circulating serum osteocalcin, supports the findings of previous studies of a negative influence of IMAT on glucose metabolism [15,16,29]. IMAT is not only a marker for age-related muscle loss; it is also linked to insulin resistance of muscle tissue. Contrarily, osteocalcin has an antidiabetic effect by enhancing insulin synthetization in the pancreas and indirectly by elevating adiponectin levels [11,12]. The exact underlying mechanism of how IMAT influences insulin resistance is still unclear, but the causes could be decreased blood flow or decreased insulin-diffusion capacity in the muscle tissue [18]. However, the exact role of osteocalcin in this context has not been clarified to date.

We could support the thesis that serum osteocalcin influences insulin levels in the male group as we could show a negative correlation between both biomarkers, suggesting a positive influence on glucose metabolism. Insulin was also negatively associated with the cross-sectional muscle area (MA). Again, no significant correlation in the female group was traceable in our data.

However, a decade ago, osteocalcin mRNA was detected in SAT and omental AT during all stages of adipogenesis, especially in the first steps, proving osteocalcin production in SAT and omental AT [30]. Whether intramuscular adipose tissue is also involved in osteocalcin production has not yet been clarified.

Blood lipid parameters and serum adiponectin levels on the other hand did not correlate significantly with MA and serum osteocalcin in both sex groups in our study population. The lack of a correlation with blood lipid parameters is in line with previous studies [31]. 

Some recent studies investigated sex differences in the axis between bone and energy metabolism before and found significant differences [32,33,34]. Serum osteocalcin levels showed significantly higher levels in the male group than in females in our study, as already described in the literature [34]. Interestingly, no association of serum adiponectin and serum osteocalcin was found in our cohort, whereas in previous studies, such an association was found mainly in women [32]. It was also assumed that osteocalcin influences insulin sensitivity in women, mainly via adiponectin [32], which could also not be confirmed for the female group in our study.

The finding that TOC is negatively related to serum insulin in our male group is in line with recent research, which could demonstrate an association between body composition using body fat quantification, TOC, and serum insulin in obese boys, but not girls [35]. The association of body fat with bone parameters, TOC, insulin, and adiponectin seem to be more sex-specific when body fat measurements with higher accuracy are used instead of BMI alone to classify obesity [35]. This could be why these results are contrary to prior research, which demonstrated negative relationships between TOC levels and serum insulin for both sexes [32]. On the other hand, this fact underlines the importance of more exact adipose tissue quantification and more exact evaluation of adipose tissue distribution. An additional explanation for the sex difference regarding insulin and TOC levels could be the different reactions of ucOC level to hypoglycemia dependent on sex [36].

Serum TOC levels decrease with age, just like muscle mass, which has been described before [37]. Interestingly our data showed this relationship only for the male population. The reason for this might be that TOC levels increase in women as late as 2 years after menopause and that circulating osteocalcin appears to be a biomarker for bone turnover in postmenopausal women [34]. However, no significant difference in circulating osteocalcin levels in postmenopausal osteoporosis patients compared to healthy controls has been demonstrated to date. Another underlying mechanism might be that the circulation of OC molecules is also influenced by glucose metabolism and could be quite heterogeneous [38]. However, further investigations should be performed to clarify this topic.

Additionally, the cross-sectional muscle area was significantly larger in our male study population than females, which is based on natural sex differences and consistent with prior studies [35]. However, our male cohort also showed significantly longer weekly training times for both endurance and strength trainingcorrelation, in turn, strongly with muscle mass.

A couple of studies investigated the effect of different exercise interventions on serum osteocalcin and glucose metabolism [10,24,39,40,41]. For example, Rahimi et al. investigated the effect of different training modalities on ucOC levels, high-molecular-weighted (HMW) adiponectin, and preptin in overweight males. They found an increase in ucOC only in the groups with concurrent aerobic interval and resistance exercise and in the group with aerobic training exercise, whereas the effect on HMW adiponectin could be seen in all training groups. However, we found a significant relation of TOC with the weekly duration of strength training in the male group. Our study subjects were predominantly healthy and of normal weight, and we only examined total osteocalcin levels, which could explain the different observations.

Dieli-Conwright et al. indicated an increase in serum osteocalcin levels in a group of female breast cancer survivors after a training intervention of 6 months [40]. In this study, the training consists of strength and endurance exercises according to a plan recommended by the American College of Sports Medicine for Cancer Survivors [42]. Interestingly, no significant relationship between the weekly training time and serum TOC levels in the female group was found in our study.

However, as Rahimi et al. highlighted in their meta-analysis, there are natural differences due to sex, the type of subjects included (healthy versus non-healthy), the type of osteocalcin measured (ucOC, carboxylated osteocalcin or TOC), and the type of training intervention [10]. The authors classified studies based on sex and demonstrated a significant rise in OC levels in studies with both men and women. However, studies including both sex groups could not demonstrate a significant change in OC. We, on the other hand, found a relationship between strength training and serum osteocalcin in the male group. When Rahimi et al. stratified studies to exercise training modalities (aerobic, resistance, and combined) in the meta-analysis, OC increased significantly when aerobic and resistance training combined was used as intervention. However, they removed two studies of which had prescribed a balanced diet along with exercise intervention, and OC levels remained unchanged in all exercise training interventions [10,41]. Therefore, the effect of additional dietary interventions remains unclear and needs further investigation. Moreover, several studies showed that TOC increases with acute bouts of exercise, such as 4 × 4 min cycling at 95% maximum heart rate high intensity interval training, which leads to glucose and free fatty acid (FFA) uptake by skeletal muscle tissue. This initiates a positive feedback circle and promotes increased glucose and FFA utilization by muscle tissue and increases the release of OCN by bone tissue [43]. One can only assume that these effects could explain the relationship between muscle mass, insulin, and TOC levels.

Additionally, recent research revealed no sex differences concerning the response of osteocalcin and its forms to high-intensity interval training [44], whereby in this study, only a short-term increase in TOC and ucOC could be observed in both sexes. Consequently, the authors emphasized the importance of regular training [44]. In contrast to results from our study, we could only demonstrate the relation of weekly resistance training, MA, and TOC in male subjects. However, the difference might be explainable by the longer training time per week in the male group.

The correlation of weekly resistance training time with serum osteocalcin suggests that, especially for men, at least a mixture of resistance and endurance training seems to have a positive effect on osteocalcin levels. This is not only essential for the maintenance of muscle mass, but it also has a positive effect on glucose metabolism. This also supports the importance of physical activity as a therapeutic measure for diabetes type 2 [45]. It also highlights the importance of physical activity to prevent age-related muscle atrophy and its consequences, especially for men [46,47].

It should also be emphasized that in our study, not all men stated that they used additional weights for training, but in some cases carried out resistance training with their body weight. However, prior studies have shown that the effect of strength training on bone mass could be increased by using additional weights [48,49].

There were some limitations in this study. The sex hormone status was not recorded, which could influence serum osteocalcin levels, especially in postmenopausal women. Additionally, as stated above, in this study, we only investigated TOC levels. Further research is planned to investigate relations of ucOC and cOC with adipose tissue distribution in different sex groups. Further, serum glucose levels have not been measured in this study, because the adequate time period between blood collection and centrifugation could not be ensured. Since this may lead to inconsistent glucose level measurements according to the literature, we refrained from glucose measurements [50]. Moreover, we are considering future studies with a closer evaluation of blood glucose levels and HOMA Index to evaluate the relationship between adipose tissue distribution and serum osteocalcin with glucose metabolism.

Another limitation is the recording of exercise with a questionnaire. In addition, the range of activity duration was very different between the subjects, which means that the standard deviation tends to be larger. Therefore, more specialized longitudinal studies, including exact performance diagnostics, should be carried out in the future.It could be mentioned, that to study the underlying mechanisms of the observed differences in more detail, animal models of obesity and physical exercise could be beneficial.

## 5. Conclusions

In summary, we were able to indicate a correlation between TOC and adipose tissue distribution, represented by the factor (SAT/(SAT + IMAT)) in the mid-thigh in the male group, which has not been described before to our knowledge. The connection of adipose tissue distribution with circulating serum osteocalcin supports the findings of previous studies, which indicate a negative influence of IMAT on glucose metabolism. Additionally, we observed a negative association between insulin and osteocalcin and the cross-sectional muscle area in males, suggesting a positive influence of osteocalcin on glucose metabolism. Above all, further longitudinal studies should be conducted to clarify the underlying mechanisms of observations made in this study and investigate the influence of strength and endurance training in this context, improved by surveying additional factors such as sex hormone status, bone density measurements, and detailed performance diagnostics. Additionally, the efficiency of bodyweight training, which has recently received much attention in fitness programmes, apps, etc., is still insufficiently studied, especially its effect on muscle mass, bone density, and insulin sensitivity, and needs further investigation.

## Figures and Tables

**Table 1:** **Descriptive statistics of the study population, including anthropometric measurements.** Areas of segmentation of cross-sectional muscle area and adipose tissues are shown along with laboratory parameters and duration of sport. Sex differences had been evaluated via Welch’s *t*-test. The shown *p*-values mark significant sex differences. (Values are reported as mean ± SD.)

Anthropometric Measurements	Total (*n* = 128)	Female (*n* = 65)	Male (*n* = 63)	*p*-Value
Age (years)	44.10 ± 11.13	46.12 ± 10.35	43.01 ± 11.59	0.090
BMI kg/m^2^)	23.87 ± 3.09	23.09 ± 3.35	24.67 ± 2.59	**0.004** **
Waist circumference (cm)	82.34 ± 10.38	77.38 ± 9.41	87.46 ± 8.77	**0.000** ***
Hip circumference (cm)	100.01 ± 7.46	98.69 ± 8.12	101.37 ± 6.51	**0.004** **
Waist/hip ratio	0.82 ± 0.08	0.78 ± 0.06	0.86 ± 0.07	**0.000** ***
Waist/height ratio	0.47 ± 0.05	0.46 ± 0.06	0.48 ± 0.05	**0.030** *
**Compartments and ratios**				
Muscle cross-sectional area (cm^2^)	282.60 ± 68.29	230.67 ± 29.13	336.18 ± 54.15	**0.000** ***
SAT (cm^2^)	5604.35 ± 2968.11	7542.11 ± 2740.52	3605.08 ± 1514.83	**0.000** ***
IMAT (cm^2^)	789.20 ± 524.67	1002.85 ± 598.84	568.76 ± 310.59	**0.000** ***
SAT/(SAT + IMAT)	0.88 ± 0.05	0.88 ± 0.05	0.87 ± 0.05	0.050
MA/IMAT	31.86 ± 42.61	16.58 ± 10.82	47.63 ± 55.68	**0.000** ***
**Laboratory measurements**				
Osteocalcin (mg/dL)	7.81 ± 3.24	6.98 ± 3.07	8.66 ± 3.22	**0.002** **
Insulin (µlU/mL)	8.30 ± 11.10	8.04 ± 11.36	8.90 ± 11.00	0.120
**Lipid profile**				
Total blood cholesterol (mg/dL)	194.13 ± 33.57	200.18 ± 30.23	187.87 ± 35.86	**0.040** *
HDL (mg/dL)	69.24 ± 17.53	75.40 ± 18.37	62.89 ± 14.16	**0.000** ***
LDL (mg/dL)	102.90 ± 31.45	102.81 ± 29.80	102.60 ± 34.39	0.970
Triglycerides (mg/dL)	112.13 ± 89.74	111.77 ± 107.48	112.49 ± 67.61	0.960
Adiponectin (µg/mL)	11.31 ± 4.12	12.36 ± 4.64	10.23 ± 3.21	**0.003** **
**Sport profile**				
Endurance (min/w)	236.52 ± 400.65	200.52 ± 436.03	273.67 ± 360.27	**0.030** *
Resistance (min/w)	83.45 ± 103.38	64.46 ± 85.81	103.05 ± 116.28	**0.002** **

Body mass index (BMI), subcutaneous adipose tissue (SAT), intramuscular adipose tissue (IMAT), muscle cross-sectional area in cm^2^ (MA), high-density lipoprotein (HDL), low-density lipoprotein (LDL); *p* < 0.05 (marked as * and **BOLD**), *p* < 0.01 (marked as ** and **BOLD**), *p* < 0.001 (marked as *** and **BOLD**).

**Table 2:** **Correlation between osteocalcin, insulin, age, tissue segmentation results, and type of sport in a sex-specific evaluation**. Pearson correlation coefficients (*r*-values) and associated *p*-values are shown.

Features	Sex	Osteocalcin (mg/dL)	*p*-Value	MA (cm^2^)	*p*-Value
Age	Female	0.19	0.138	−0.18	0.152
	Male	**−0.31**	**0.014** *	**−0.32**	**0.000** ***
SAT/(SAT + IMAT)	Female	0.12	0.343	0.21	0.100
	Male	**0.27**	**0.029** *	**0.38**	**0.002** **
IMAT/(SAT + IMAT)	Female	−0.12	0.343	−0.21	0.100
	Male	**−0.27**	**0.029** *	**−0.38**	**0.002** **
MA/IMAT	Female	0.04	0.729		
	Male	0.23	0.075		
MA/(IMAT + MA)	Female	0.10	0.427		
	Male	0.21	0.097		
Muscle cross-sectional area (cm^2^)	Female	−0.09	0.486		
	Male	**0.26**	**0.038** *		
Strength training (min/w)	Female	−0.08	0.519	0.24	0.059
	Male	**0.30**	**0.019** *	**0.38**	**0.000** ***
Endurance sports (min/w)	Female	−0.00	0.983	0.23	0.065
	Male	0.14	0.259	0.21	0.017 *
Insulin (µlU/mL)	Female	0.16	0.218	−0.02	0.375
	Male	**−0.32**	**0.010** *	**−0.28**	**0.033** *
Adiponectin (µg/mL)	Female	−0.17	0.893	−0.09	0.482
	Male	0.06	0.647	−0.08	0.552
Total blood cholersterol (mg/dL)	Female	−0.08	0.523	−0.11	0.381
	Male	−0.16	0.203	−0.04	0.751
HDL (mg/dL)	Female	−0.13	0.308	−0.05	0.681
	Male	0.03	0.835	0.01	0.951
LDL (mg/dL)	Female	0.20	0.124	−0.07	0.599
	Male	−0.09	0.491	−0.01	0.927
Triglycerides (mg/dL)	Female	−0.03	0.217	−0.05	0.664
	Male	0.24	0.062	−0.10	0.436

Subcutaneous adipose tissue (SAT), intramuscular adipose tissue (IMAT), muscle cross-sectional area in cm^2^ (MA); *p* < 0.05 indicated as (*), *p* < 0.01 (**), *p* < 0.001 (***). Statistically significant values are written in **BOLD**.

## Data Availability

The data presented in this study are openly available in Fig Share at https://doi.org/10.6084/m9.figshare.17060237 (accessed on 22 November 2021).
